# Effects of Periodontitis on Major Organ Systems

**DOI:** 10.7759/cureus.46299

**Published:** 2023-09-30

**Authors:** Drishti V Lohiya, Ashok M Mehendale, Divya V Lohiya, Harsh S Lahoti, Vidhi N Agrawal

**Affiliations:** 1 Preventive Medicine, Jawaharlal Nehru Medical College, Datta Meghe Institute of Higher Education and Research, Wardha, IND; 2 Otolaryngology, Jawaharlal Nehru Medical College, Datta Meghe Institute of Higher Education and Research, Wardha, IND

**Keywords:** reproductive systems, central nervous, musculoskeletal, endocrine, pulmonary, cardiovascular, dental health, systemic diseases, periodontitis, oral health

## Abstract

This review focuses on the fact that oral disorders are not merely localized to the mouth; in a broader sense, they also have a more significant impact on systemic health. In this review, we tried to bring to the notice various complications of periodontitis on the body's major organ systems. It has also been suggested that there is a potential connection between periodontitis and certain systemic disorders. Reviewing this fascinating topic is necessary. The objective is to create a thorough body of knowledge on the subject that is simple to access, alert medical professionals to the connection between dental health and systemic health, and highlight the necessity of a more thorough incorporation of medical and dental training. Periodontitis is a probable risk factor for various problems connected to the cardiovascular, pulmonary, endocrine, musculoskeletal, central nervous, and reproductive systems. It is a continual likely source of infection. Oral health affects overall health, and if extensive healthcare is ever accomplished, dental health should never be considered a distinct, remote, and lower significant part of health wholly disconnected from quality of life. One should never underestimate oral disorders as being acute and always curable. People should take utmost care and take the condition seriously to prevent significant complications.

## Introduction and background

The periodontal ligament, alveolar bone, cementum, and gingiva form a supporting framework of the teeth. When the periodontal condition has advanced past gingivitis, periodontitis, a tenacious, detrimental, and enduring infectious illness, aggravates. The microorganisms can then further colonize the tissues along with the periodontium. To fight the invasive organism, the host responds. However, in defending against the bacteria, the host defenses lead to the demolition of the periodontium. Periodontitis causes the periodontium to lose the attachment, leading to alveolar bone loss and may ultimately lead to the loss of the affected tooth [1]. Research on the topic suggests that the chronic inflammatory conditions of the tooth-supporting structures known as periodontitis lead to an elevated risk of mortality or morbidity for numerous systemic diseases, for example, diabetes mellitus, spontaneous preterm birth (SPB) and various cardiac conditions (atherosclerosis, heart attack, and stroke) [2]. Recent case-control and cross-sectional studies suggest that periodontitis can aggravate cardiovascular disease risk by approximately a factor of two and preterm low birth weight neonates risk about by a factor of seven, respectively [3]. 

Several periodontopathic bacterial species can be demonstrated in pyogenic liver abscess, some of them as Fusobacterium nucleatum, Treponema denticola, Prevotella intermedia, and Porphyromonas gingivalis, various unusual autopsy cases of pyogenic liver abscess which is as a result of infection by periodontal bacteria are highly accounted [4]. All alterations in systemic markers are thought to be caused by possible daily occurrences of bacteremia from periodontal lesions [5]. It is still unclear how oral bacteria such as Porphyromonas gingivalis influence the pathogenesis of Alzheimer's disease and their potential causal role [6]. 

According to recent research, the relationship between periodontal infections and COVID-19 can be described by the direct contribution of a periodontal microbe to the exacerbation of respiratory lung diseases and by the indirect effects of Periodontitis on systemic inflammation and immune system primarily leading to an intensified reciprocation to severe acute respiratory syndrome infection with coronavirus 2 [7]. A powerful periodontal bacteria called P. gingivalis can enter the bloodstream and cause a prothrombotic condition. P. gingivalis expresses a large number of virulence factors that help the organism to infect a range of host systems and cells, avoid spotting by the host immune system, trick the host immune system into producing the wrong antibodies, and activate several humoral and cellular hemostatic mechanisms [8].

## Review

Chronic periodontitis

Chronic periodontitis, also called adult periodontitis, is an infectious inflammatory condition caused by the bacteria in dental plaque. It leads to the tissues that hold up the teeth gingiva, the periodontal ligament, the cementum, and the alveolar bone gradually deteriorating [9,10]. The preliminary and basic causative factors that result in periodontal infection are the assorted bacterial colonization in the oral tissue [11]. Major pathogens causing periodontitis are Aggregatibacter actinomycetecomitans, Porphyromonas gingivalis, Bacteroides forsythias, Prevotella intermedia, Campylobacter rectus, Treponema denticola, Fusobacterium nucleatum, many more [12,13]. At the same time, additional elements like cervical enamel projections, short trunks, calculus, plaque from teeth, overhanging restorations, developmental grooves, smoking, stress, and inherited characteristics act as supplementary etiological variables speeding up the progression and progression of periodontal diseases [14,15]. 

Chronic inflammatory illnesses with a high incidence include gingivitis and chronic periodontitis. Most individuals have gum disease, and 5-15% of adults are believed to suffer from advanced periodontitis. One of the essential components of oral health care is recognizing and determining the cause of these prevalent conditions. Each individual should have a regular oral evaluation, including a periodontal appraisal. All newly diagnosed patients should undergo a periodontal screening as part of continuous oral medical care, utilizing techniques like the basic periodontal examination/community periodontal index or periodontal screening record. If periodontitis has been identified, an extensive periodontal investigation is required. This includes collecting whole mouth probe and hemorrhage data and assessing extra relevant factors such as plaque levels, furcation involvement, and recession. A clinical and radiographic evaluation is necessary for aggressive and chronic periodontitis patients. The clinical examination comprises of estimation of local factors, determining furcation involvement, recession, estimating periodontal pocket depth using pressure sensitive probes or manually, determining to bleed on probing (a sign of periodontal tissue inflammation), and recognizing dental variations in development, which result in additional plaque piling up [16]. 

**Figure 1:**
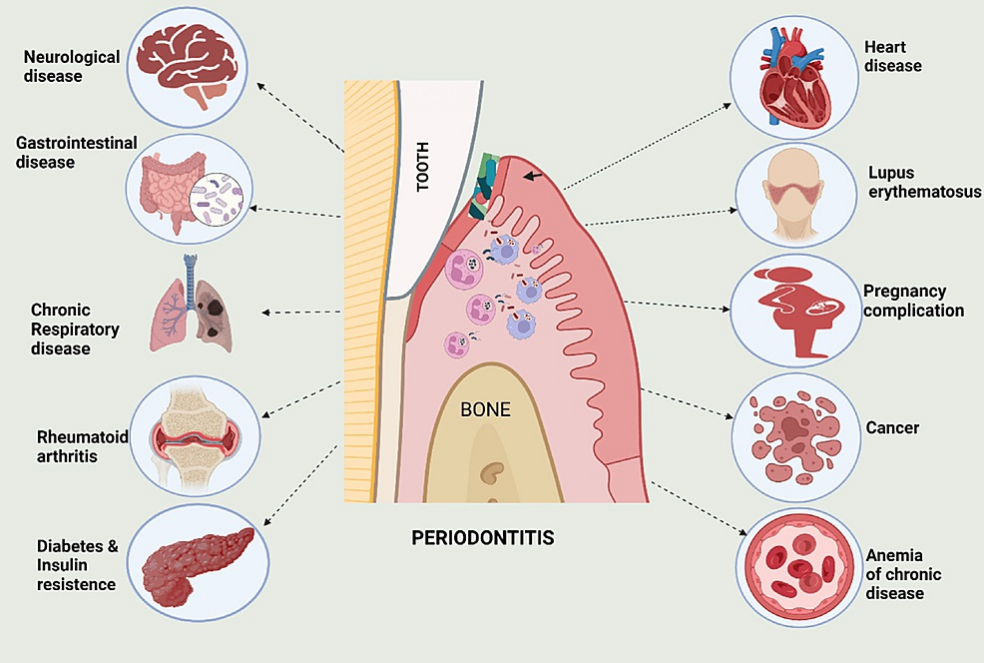
Compilation of effects of periodontitis on major organ systems This image is obtained from an open access journal [18].

To evaluate bone deterioration among people with periodontitis, radiographic evaluation of the alveolar bone levels is needed. The clinical condition influences this evaluation. A vital component of dental treatment should involve risk assessment and risk management, such as encouraging quitting smoking and determining the presence of diabetes. The conventional clinical features, along with the radiographic assessment, determine the presence of aggressive periodontitis [16]. A case history, physical examination, and radiological analysis are employed to identify severe periodontitis. These techniques are prone to fairly significant measurement mistakes in the acquired data. In addition, this diagnostic technique only analyses the previous phase of the sickness; it may not be sufficient to accurately identify present disease progression or correctly forecast potential tissue loss. A diagnosis often occurs years after the disease initially emerges, partly because incipient or initial lesions, where tissue loss is limited and generally below the threshold for detection for present examination methods, are more unlikely to be identified through current evaluation techniques than developed lesions. Further advances in our comprehension of the cause of this illness may assist us in establishing a diagnosis earlier. Mirror-like arch deformities can be noticed in the area of the permanent molars on an oral pantograph [17]. Figure 1 demonstrates a compiled representation of how periodontitis affects various organ systems [18].

Association with major organ systems

Cardiovascular Systems

Chronic periodontitis is related to the occurrence of coronary heart diseases in the youth population, irrespective of traditional cardiovascular risk factors [19]. According to a developing body of epidemiologic, in vitro, clinical, and animal studies, periodontal infection may indicate a risk for heart disease. However, significant concerns have been raised about the exact purpose of this link. These are initial research. More studies must be undertaken to determine this connection since a slight possibility of periodontal disease triggering heart disease could result in significant death or disability. Assessing any possible statistically noticeable reduction in heart disease brought by either the avoidance or treatment for periodontal disease is one essential topic yet to be worked across. Possibly linked to myocardial infarction is subgingival periodontal pathogenic infection. Since carotid atheromas have been linked to oral bacteria and some oral bacteria can be linked to platelet aggregation, a crucial step in thrombosis, basic laboratory investigations indicate that this association is biologically plausible [20]. 

Atherosclerosis-related cardiovascular disease (ASCVD) and periodontitis are frequent chronic inflammatory conditions. Periodontitis-induced systemic inflammation may cause hematopoietic stem and progenitor cells (HSPCs) to adapt, leading to assisted granulopoiesis in the bone marrow. This could boost the formation of neutrophils and favor the hyperresponsiveness of numerous innate immune cells. These changes can impact atherosclerosis's development, progression, and consequences [21]. Independent of other confounding factors, periodontal disease raises C reactive protein levels in individuals with acute myocardial infarction, becoming an additional goal for minimizing potential risks in acute myocardial infarction (AMI) survivors [22]. 

Respiratory System

Nosocomial infections, particularly those related to the urinary system, surgical wounds, and pneumonia, are the major cause of mortality in critically sick individuals in intensive care units (ICU). For respiratory viruses linked to hospital-acquired pneumonia, ICU patients' mouths can act as a significant reservoir [23]. Multiple studies discovered that individuals with substandard oral hygiene have a nearly fivefold higher risk of developing chronic respiratory disorders than those with adequate oral hygiene. Lung infections, particularly nosocomial pneumonia episodes in high-risk people, are influenced by poor dental hygiene and periodontitis [24]. The dental cavity had frequently been suspected as a major cause of respiratory infections. Various infection methods might involve aspiration of pneumonia-causing oral pathogens into the lung colonization of dental plaque by respiratory pathogens, which is then accompanied by aspiration or encouragement of colonization of the upper airway by pulmonary and periodontal pathogens [25]. Several systemic disorders, such as diabetes, obesity, atherosclerotic heart disease, Alzheimer's disease, and problems linked to other diseases, are all related to poor oral health. In addition, lung infection can result from oral bacteria colonization of the lower respiratory tract and alteration of the mucosal surface; both aggregate the deterioration of the lung epithelial surface and form a favorable condition for critical COVID-19 infection. Periodontal disease has been highly related to the elevated risk of gaining critical COVID‐19 infection, hospitalization, and mortality [26].

Musculoskeletal System

The risk of developing rheumatoid arthritis increases in individuals with moderate to severe periodontitis, as found in studies. Although there may be differences in the etiology of both these diseases, the basic pathogenic mechanisms are strikingly the same, and it is found that people who have periodontitis and rheumatoid arthritis both might be affected by a common systemic rearrangement of the inflammatory response [27]. Bone metabolism is significantly regulated by the gut microbiota [28]. A significant population-based investigation revealed that osteoporosis was more prevalent in the genera Bacteroides, Blautia, Phascolarctobacterium, Oscillospira, Ruminococcaceae, and Actinobacillus [29]. The entry point in the digestive system/gastrointestinal tract, the oral cavity, harbors a variety of microbial communities [30]. Earlier studies have provided evidence in favor of the idea that the gastrointestinal and oral mucosae are related microbiologically [31,32]. The periodontitis-related oral pathobiont-rich salivary microbiota may also translocate to the intestine and affect the colonization resistance of gut-dwelling microbiota [33].

Reproductive System

Research and studies proved the notable relationship between preterm birth and low birth weight and Periodontitis, regardless of parity, race, and maternal age [34]. Preterm or spontaneous preterm deliveries are more likely in mothers with periodontal disease. Additionally, despite conventional obstetric, periodontal, and social domain risk factors, the progression of periodontal disease throughout pregnancy predicts a more severe pregnancy outcome of preterm birth [35]. It is supposed to happen because bacterial infection triggers the release of cytokines that have been associated with the mechanism of labor, including interleukins (IL) IL-1, IL-6, tumor necrosis factor (TNF-a), and prostaglandins (PDE2). According to a recently hypothesized mechanism of labor, these mediators' intra-amniotic levels increase steadily throughout pregnancy until they reach a critical level, which triggers the onset of labor. As an outcome, it increases the probability that infection leads to an exceptionally significant release of physiological mediators of parturition that might result in birth and lead to low birth weight. Additionally, as assumed that subclinical infections like periodontal disease lead to preterm labor and low birth weight by inducing the release of cytokines in the deciduas or membranes, which in turn leads to an increase in prostaglandin levels or even uterine muscle contraction [36,37]. According to recent studies, gestational diabetes mellitus is more likely to develop in pregnant women with periodontal disease than in pregnant women with healthy gums [38]. 

Endocrine System

Given the fact that individuals with diabetes are more prone to develop periodontal disease, recent studies indicate that diabetes might also increase the probability of periodontal disease [38]. Bacteria enter the bloodstream secondary to the consequence of periodontal disease, causing immune cells to become active. The latest study has demonstrated that elevated cholesterol levels may be among the root causes of the immune cell changes put on by diabetes. Recent studies on people have associated high levels of lipids in the blood and Periodontitis. Some evidence supports the notion that Periodontitis may cause higher low-density lipoprotein (LDL)/ triglycerides (TRG) levels. An increase in serum proinflammatory cytokines, which include interleukin-1 beta (IL-1 beta) and tumor necrosis factor-alpha (TNF-alpha), which are found to create modifications to the metabolism of lipids leading to high levels of lipids have been associated with periodontitis-induced bacteremia/endotoxemia. In this instance, Periodontitis may be related to higher levels of inflammatory agents, proinflammatory cytokines, and serum lipids, as well as a possible systemic illness caused by persistent hyperlipidemia and/or increased inflammatory mediators. Inflammatory biological signals (cytokines) are formed by these stimulated cells, which are known to hurt the whole body system [39]. The pyogenic granuloma (PG) is a reactive growth that develops as an inflammatory reaction to local irritation from conditions like periodontitis, calculus, a broken tooth, abrasive dental repair, foreign objects, or hormonal (pregnancy tumor) and it is sometimes linked to bone loss. The pancreatic cells that form insulin have been proven to be harmed or destructed by chronically elevated levels of cytokines. Additionally, once this happens, it may result in Type 2 diabetes in otherwise healthy people with no additional risk factors for the disease [40,41]. Diabetes is affected by periodontitis in terms of prevalence, progression, and therapeutic management. The metabolic condition of a diabetic may be significantly impacted by periodontal disease. Recent research results suggest that treating periodontal disease may help to improve glycemic management [42].

Renal System

Although there is strong evidence between chronic kidney disease (CKD) and periodontal infection (PO), several studies suggest an opposite correlation between the two illnesses. The analysis is done on the strong and potential link between chronic kidney disease and periodontal disease. The main pathophysiology that connects both these diseases is addressed as systemic inflammation, endothelial dysfunction, and variance of oxidative stress that are hallmarks of chronic kidney disease that may affect the oral microbiota's escape mechanisms and play an important part in periodontitis development. Subclinical regional and systemic inflammation brought on by PO may impact the course of CKD [43]. Periodontitis is an emerging risk factor for advanced CKD8 that may be managed. The endothelial function of the nephrons may be affected by the systemic migration of periodontal infections through circulation [44]. Furthermore, periodontitis can change the body's equilibrium by releasing endotoxins and inflammatory cytokines [45]. Much evidence shows middle-aged and older persons with generalized periodontitis and severe CKD stage 3 are related [46]. A public health concern with an estimated global incidence of 13.4% is chronic kidney disease (CKD), which is marked by a decreased estimated glomerular filtration rate (eGFR) and the existence of microalbuminuria or proteinuria. Initial-stage CKD patients can go years without exhibiting signs or symptoms, and only at greater degrees do individuals start to experience the typical kidney damage issues. For people with advanced CKD, Periodontitis is a new risk factor that could be managed. The systemic translocation of periodontal disease across the circulation may affect nephron endothelial function. Through the discharge of endotoxins and inflammatory cytokines, periodontitis may influence the homeostasis of the entire body. Localized periodontitis associated with systemic inflammation may be a factor in young adults' acute or chronic kidney injury, evidenced by proteinuria, especially in those with a modestly reduced glomerular filtration rate [47,48].

Central Nervous System

The most prevalent form of dementia in older individuals is Alzheimer's disease (AD), the most prevalent cause of dependency and impairment in this stage of life. However, there is a youth-afflicted category; it is more common in women than men and occurs more often in adulthood beyond the age limit of 65 [49]. It was recently hypothesized that systemic diseases might be essential in inducing the brain and spinal cord to get inflamed. In this regard, periodontal infections are one of the most widespread oral cavity infections in adults, as well as the most prevalent oral disease in individuals, and an important factor contributing to tooth loss in the elderly [50,51]. It is caused by the growth of a pathobiont- or keystone pathogen-produced dysbiotic microbiota. These microbes or their virulence factors can cause harm directly or indirectly to the periodontal tissues by triggering the host's immune system, leading to the development of a subgingival pocket, chronic inflammation of the periodontal tissues, and erosion of alveolar bone. By triggering the immune response in the host and increasing the production of proinflammatory mediators such as interleukin (IL)-1, IL-1, IL-6, tumor necrosis factor (TNF), prostanoids as well as matrix metalloproteinases (MMPs), that possess the possibility for dissemination to the bloodstream, this dysbiotic microbiome has the capability of causing a low-grade systemic inflammatory response. The dysbiotic microbiota regulates the breakdown of bones and the degeneration of periodontal connective tissue at the local level. It makes the host sensitized to multiple cytokines and inflammatory mediators on the systemic level. The endotoxins and lipopolysaccharide (LPS)-rich bacteria linked to Periodontitis encourage the proliferation of immune system cells and the generation of cytokines. IL-1, IL-6, and TNF- are transported to the central nervous system via circulation or peripheral nerve terminals. Cytokines, microbes, or their virulence factors can ultimately excite glial cells and produce neuroinflammation after reaching the brain, which may assist in developing or worsening AD [49].

The effect of periodontitis on various organs and its disorders are briefly described given in Table 1.

**Table 1:** Correlation of periodontitis with various systemic disorders. COVID-19: Coronavirus disease 2019; LDL: low-density lipoprotein; TRG: triglycerides.

Organ System	Periodontal Correlation
Cardiovascular System	Coronary heart disease, Atherosclerosis, Heart Attack, Clotting problems in Cardiovascular System
Respiratory System	Lower Respiratory tract infections, Nosocomial Pneumonia, Critical COVID-19
Musculoskeletal System	Rheumatoid Arthritis, Osteoporosis, Osteopenia
Reproductive System	Preterm Birth, Low birth weight, Gestational Diabetes Mellitus, Crossing the Placental Barrier lead to Fetal Infections
Endocrine System	Uncontrolled Type 2 Diabetes Mellitus, High Levels of LDL/TRG level, Pyogenic Granuloma
Renal System	Acute and Chronic Kidney Disease
Central Nervous System	Alzheimer’s Disease, Stroke

## Conclusions

One's overall well-being may be affected by their oral health, either directly or indirectly. Medical personnel must realize the fact's increasing significance of delivering complete medical care. Dentists need to expand their clinical experience to relevant systemic illnesses and their clinical competence of these ailments to interact and relate to their medical colleagues successfully. Following current knowledge, regular dental examinations are highly recommended. One should keep in check about oral hygiene as negligence it may lead to systemic complications and affect the major body systems. 
